# Delirium in Children Undergoing Hematopoietic Cell Transplantation: A Multi-Institutional Point Prevalence Study

**DOI:** 10.3389/fonc.2021.627726

**Published:** 2021-04-22

**Authors:** Chani Traube, Linda M. Gerber, Elizabeth A. Mauer, Keshia Small, Larisa Broglie, Yogi Raj Chopra, Christine N. Duncan, Christen L. Ebens, Julie C. Fitzgerald, Jason L. Freedman, Michelle P. Hudspeth, Caitlin Hurley, Kris M. Mahadeo, Jennifer McArthur, Miriam C. Shapiro, Matthew P. Sharron, Donna A. Wall, Matt S. Zinter, Bruce M. Greenwald, Gabrielle Silver, Farid Boulad

**Affiliations:** ^1^Department of Pediatrics, Weill Cornell Medical College, New York, NY, United States; ^2^Department of Pediatrics, MSK Kids at Memorial Sloan Kettering Cancer Center, New York, NY, United States; ^3^Department of Population Health Sciences, Weill Cornell Medical College, New York, NY, United States; ^4^Department of Medicine, Weill Cornell Medical College, New York, NY, United States; ^5^Department of Pediatric Hematology/Oncology/Stem Cell Transplantation, Columbia University Medical Center, New York, NY, United States; ^6^Department of Paediatrics, The Hospital for Sick Children, University of Toronto, Toronto, ON, Canada; ^7^Department of Pediatric Hematology/Oncology, Dana-Farber Cancer Institute, Boston, MA, United States; ^8^Department of Pediatrics, University of Minnesota, Minneapolis, MN, United States; ^9^Department of Anesthesiology and Critical Care, Children's Hospital of Philadelphia, University of Pennsylvania Perelman School of Medicine, Philadelphia, PA, United States; ^10^Department of Pediatrics, Children's Hospital of Philadelphia, University of Pennsylvania Perelman School of Medicine, Philadelphia, PA, United States; ^11^Department of Pediatrics, Medical University of South Carolina, Charleston, SC, United States; ^12^Department of Bone Marrow Transplant & Cellular Therapy, St Jude Children's Research Hospital, Memphis, TN, United States; ^13^Department of Stem Cell Transplantation and Cellular Therapy, Children's Cancer Hospital, University of Texas at MD Anderson Cancer Center, Houston, TX, United States; ^14^Department of Pediatrics, Children's National Hospital, George Washington University School of Medicine, Washington, DC, United States; ^15^Department of Pediatrics, University of California, San Francisco, San Francisco, CA, United States; ^16^Department of Psychiatry, Weill Cornell Medical College, New York, NY, United States

**Keywords:** cancer, pediatric oncology, hematopoietic cell transplant, delirium, cornell assessment of pediatric delirium, incidence, risk factors

## Abstract

**Introduction:** Delirium occurs frequently in adults undergoing hematopoietic cell transplantation, with significant associated morbidity. Little is known about the burden of delirium in children in the peri-transplant period. This study was designed to determine delirium rates, define risk factors (demographic and treatment related), and establish feasibility of multi-institutional bedside screening for delirium in children undergoing hematopoietic cell transplant.

**Methods:** This is a multi-institutional point prevalence study. All subjects were prospectively screened for delirium twice daily using the Cornell Assessment of Pediatric Delirium over a 10-day period. De-identified data, including basic demographics and daily characteristics, were extracted from the electronic medical record.

**Results:** Eleven North American institutions were included, 106 children were enrolled, and 883 hospital days were captured. Delirium screening was successfully completed on more than 98% of the study days. Forty-eight children (45%) developed delirium over the course of the 10-day study. Children were diagnosed with delirium on 161/883 study days, for an overall delirium rate of 18% per day. Higher delirium rates were noted in children <5 years old (aOR 0.41 for children over 5 years), and in association with specific medications (melatonin, steroids, and tacrolimus).

**Conclusion:** Delirium was a frequent occurrence in our study cohort, with identifiable risk factors. Delirium screening is highly feasible in the pediatric hematopoietic cell transplant patient population. A large-scale prospective longitudinal study following children throughout their transplant course is urgently needed to fully describe the epidemiology of pediatric delirium, explore the effects of delirium on patient outcomes, and establish guidelines to prevent and treat delirium in the peri-transplant period.

## Introduction

Delirium is a frequent complication of serious pediatric illness, with an incidence >25% in the pediatric intensive care unit (PICU) (1–3). Delirium is defined as an acute and fluctuating syndrome, and includes altered awareness and cognition. It occurs as a result of an underlying medical condition or as a side effect of treatment for that condition (4). Studies have shown that children diagnosed with delirium have increased short- and long-term morbidity, and even excess mortality (5–7). Modifiable iatrogenic risk factors have been identified for delirium in critically ill children, and changes in treatment approaches have led to lower delirium rates in the PICU (8–10).

Delirium is also a well-known complication of hematopoietic cell transplantation (HCT) in adults, affecting >50% of patients in the 4 weeks after transplant (11, 12). In these adults, delirium is associated with increased mortality (both in-hospital and up to 5 years after discharge) and substantial morbidity, including increased use of opioid analgesics, increased hospital length of stay, and increased family and healthcare team distress. In HCT survivors, delirium has been associated with decreased performance status upon discharge, increased anxiety disorders, and effects on neurocognitive ability that persist at 1 year after discharge (13–16). As a result, units have adopted changes in approach to caring for adults after transplant, which have resulted in a decrease in delirium rates (17). However, little is known about the burden of delirium in children undergoing HCT. As a result of this critical knowledge gap, no similar changes have taken place in pediatric HCT programs (18).

Our objectives in this multi-institutional point prevalence study were (i) to determine delirium rates over a 10-day period in children undergoing HCT, (ii) to explore risk factors (both demographic and treatment related) associated with the diagnosis of delirium, and (iii) to establish the feasibility of multi-institutional bedside screening for delirium in children undergoing HCT. We hypothesized that delirium rates would exceed 25% in these high-risk children. We further hypothesized that prescribing characteristics (for blood products and medication categories) would differ significantly between patients with and without delirium, and prescribing practices would also vary substantially among participating sites; this could present a possible opportunity to modify practices. Additionally, we hypothesized that delirium screening would be feasible in all units, with a daily screen completion rate of >80%.

## Materials and Methods

### Study Design

This is a multi-institutional prospective point-prevalence study. Sites were invited to participate *via* emails sent to members of the following research consortiums: the HCT subgroup of the Pediatric Acute Lung Injury and Sepsis Investigators (PALISI), and the Pediatric Bone Marrow Transplant Consortium Supportive Care Strategy Group. All interested sites were included. Study personnel at each site completed an online training session and test in order to certify for study participation. The study took place over the same 10-day period in April 2019. All patients 0–21 years old who had been admitted to the pediatric service for allogeneic or autologous stem-cell transplants were eligible for inclusion, regardless of timing—we included all children during conditioning, transplant, and engraftment. Children were excluded if they had been admitted for reasons other than transplant (for example: delayed complications after transplant), as the purpose of this study was to specifically describe delirium in the peri-transplant period.

**Figure 1:**
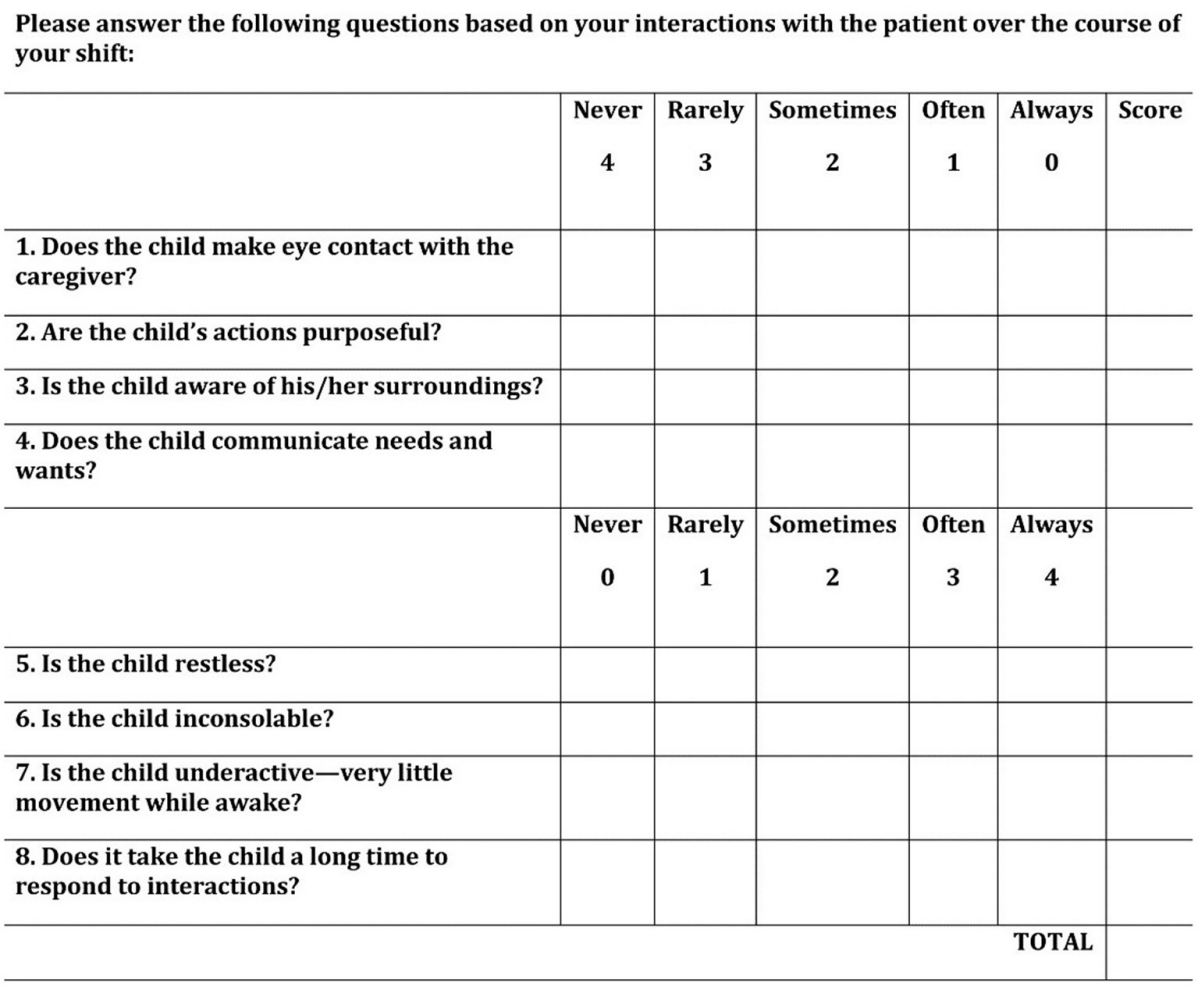
Cornell Assessment for Pediatric Delirium (CAPD). The CAPD is a rapid bedside screening tool validated for delirium detection in children of all ages. A score of nine or higher is consistent with a diagnosis of delirium, and has been shown to correlate with patient outcome measures. [Reproduced from reference (19), with permission of Wolters Kluwer Health].

All subjects were screened for delirium twice daily by their nurses, ~12 h apart, during the 10-day study period using the Cornell Assessment for Pediatric Delirium (CAPD) (Figure 1) (19). The CAPD is a bedside tool that has been validated for use in children of all ages and developmental stages. It is an observational tool that consists of eight items, requires <2 min to complete, and is designed to be scored by the bedside nurse at the end of each shift. The scoring process is the same in children of all ages, but a developmental anchor point chart is available for use as a point-of-care reference (to remind the nurse what behaviors to expect in pre-verbal children) when scoring the CAPD in children <2 years (20). Operational definition of delirium was a CAPD score of nine or higher (19).

In addition, a brief data collection form (with no patient health information included) was completed for each patient. The de-identified data included basic demographics, diagnosis, type and timing of transplant, conditioning regimen, respiratory support, daily medication classes, and delirium scores. The medications were captured on a daily basis to allow for assessment of the temporal relationship between medication exposure and delirium development. Data were entered into an electronic case report form within the Research Electronic Data Capture (REDCap) system, a secure centralized database hosted at the data coordinating center, Weill Cornell Medical College (WCMC) (21). Each site received local Institutional Review Board (IRB) approval, with waiver of informed consent for this minimal risk observational study.

### Statistical Analysis

Patient-level demographic, clinical, and transplant characteristics were described as counts and percentages (*n*, %), mean and standard deviation (sd), or median and range [min, max, or interquartile range (IQR)]. Delirium was defined at the patient level as any delirium (CAPD score of nine or higher) during the days captured. Bivariate analyses compared those subjects who were delirious with those subjects who were never delirious, using Chi-square/Fisher's exact tests, or independent two-sample *t*-tests/Wilcoxon rank-sum tests as appropriate. A logistic mixed effects regression model was used to assess multivariate associations with delirium, controlling for patient age, type of transplant (allogeneic vs. autologous), and diagnosis. A random intercept for site was included to account for within-site correlation among patients.

Characteristics of each hospital day were also described, as *N* (%) or mean (sd), median (min, max, IQR). A day was characterized as “delirium” if the CAPD score was nine or higher on either or both of the daily screens. Bivariate relationships with daily delirium were analyzed as described above. Similarly, a logistic mixed effects regression modeled the association between delirium and predictor variables (transplant day, respiratory support, and receipt of specific medication categories), with a random intercept for site to account for within-site correlation among hospital days. Independent variables included in the multivariate model are presented with adjusted odds ratios and the associated 95% confidence intervals (CI). All *p*-values were two sided with statistical significance evaluated at the 0.05 alpha level. Analyses were performed in R version 4.0.2 (R Foundation for Statistical Computing, Vienna, Austria).

## Results

### Participating Sites

Over the course of a 10-day period in April 2019, 11 institutions participated in this point prevalence study. Five of the participating sites were dedicated cancer hospitals, and five were dedicated children's hospitals; one was a pediatric transplant unit housed within a larger mixed-use hospital. Only two sites had a standard-of-care requirement for routine delirium testing prior to this study. Among the sites, the median reported number of hematopoietic cell transplants each year is 70 (IQR 45–100); 65% of these are allogeneic.

### Subjects

One hundred six children were eligible and included in this study. Fifty-nine percent of the study cohort were males. The patients' median age was 4 years, and 45% of the children were under the age of 5 years. Indications for transplant and subject characteristics are listed in Table 1. Seventy percent of the children had malignancies. Sixty-six percent of the children underwent allogeneic hematopoietic cell transplants. Of the 90 children (85%) who underwent myeloablative conditioning, 15 (17%) had total body irradiation. For graft-versus-host-disease (GVHD) prophylaxis, 49% of the children received calcineurin inhibitors.

**Table 1:** Demographic and clinical characteristics of study cohort (*n* = 106).

	**Overall**
	***n* (%)**
**Age**	
0– <1 year	12 (11.3%)
1– <2 years	6 (5.7%)
2– <5 years	30 (28.3%)
5– <13 years	28 (26.4%)
13–21years	30 (28.3%)
**Sex**	
Male	63 (59.4%)
Female	43 (40.6%)
**Diagnosis**	
Leukemia/lymphoma	40 (37.7%)
Solid tumor	29 (27.4%)
Primary immunodeficiency	14 (13.2%)
Metabolic disorder	7 (6.6%)
Aplastic anemia/inherited bone marrow failure	6 (5.7%)
Myelodysplastic syndrome	4 (3.8%)
Hemoglobinopathy	3 (2.8%)
Other	3 (2.8%)
**Transplant type**	
Allogeneic	70 (66%)
Autologous	36 (34%)

### Hospital Days

**Figure 2:**
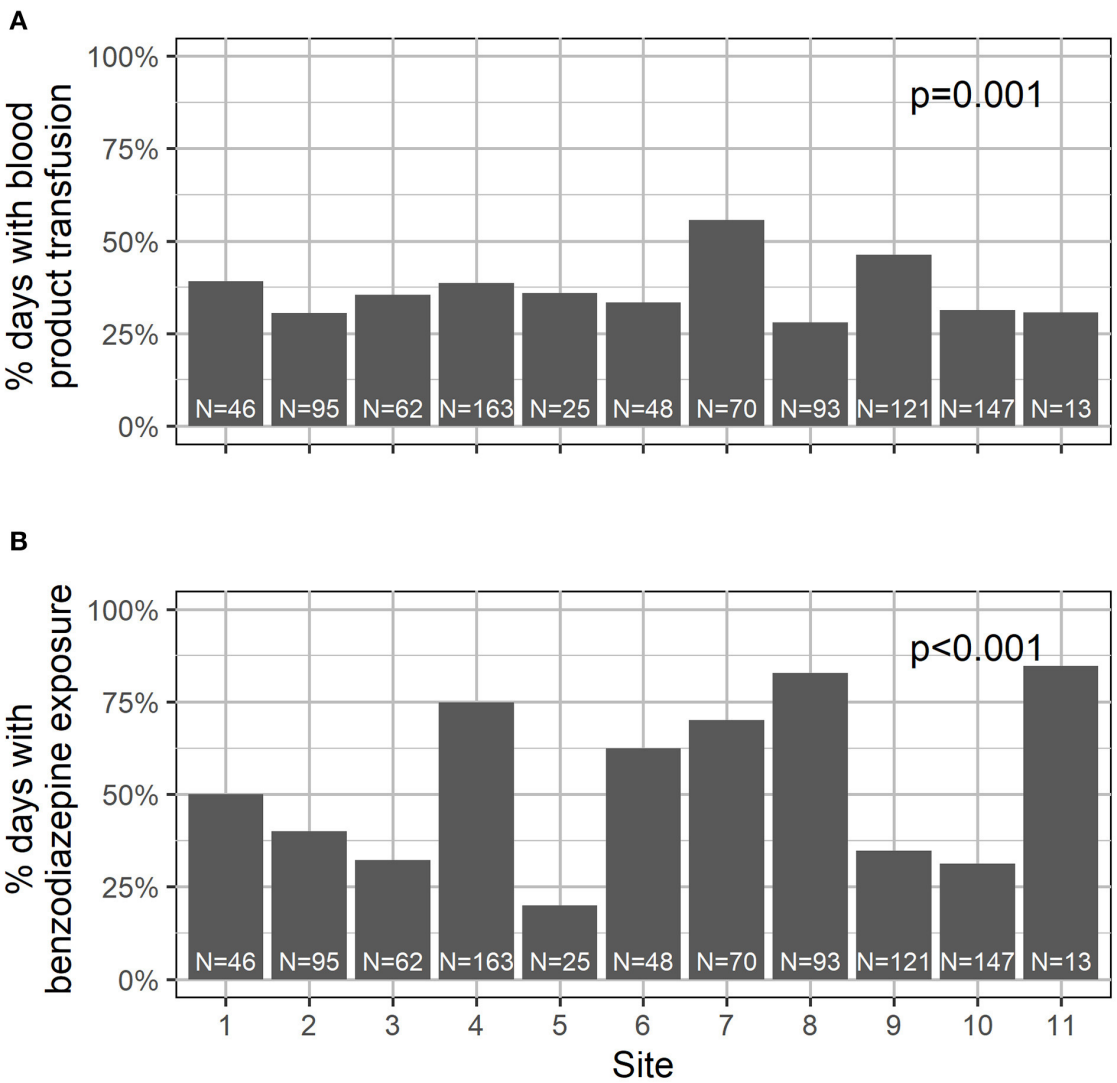
Transfusion (**A**) and benzodiazepine (**B**) use differed significantly by site.

**Figure 3:**
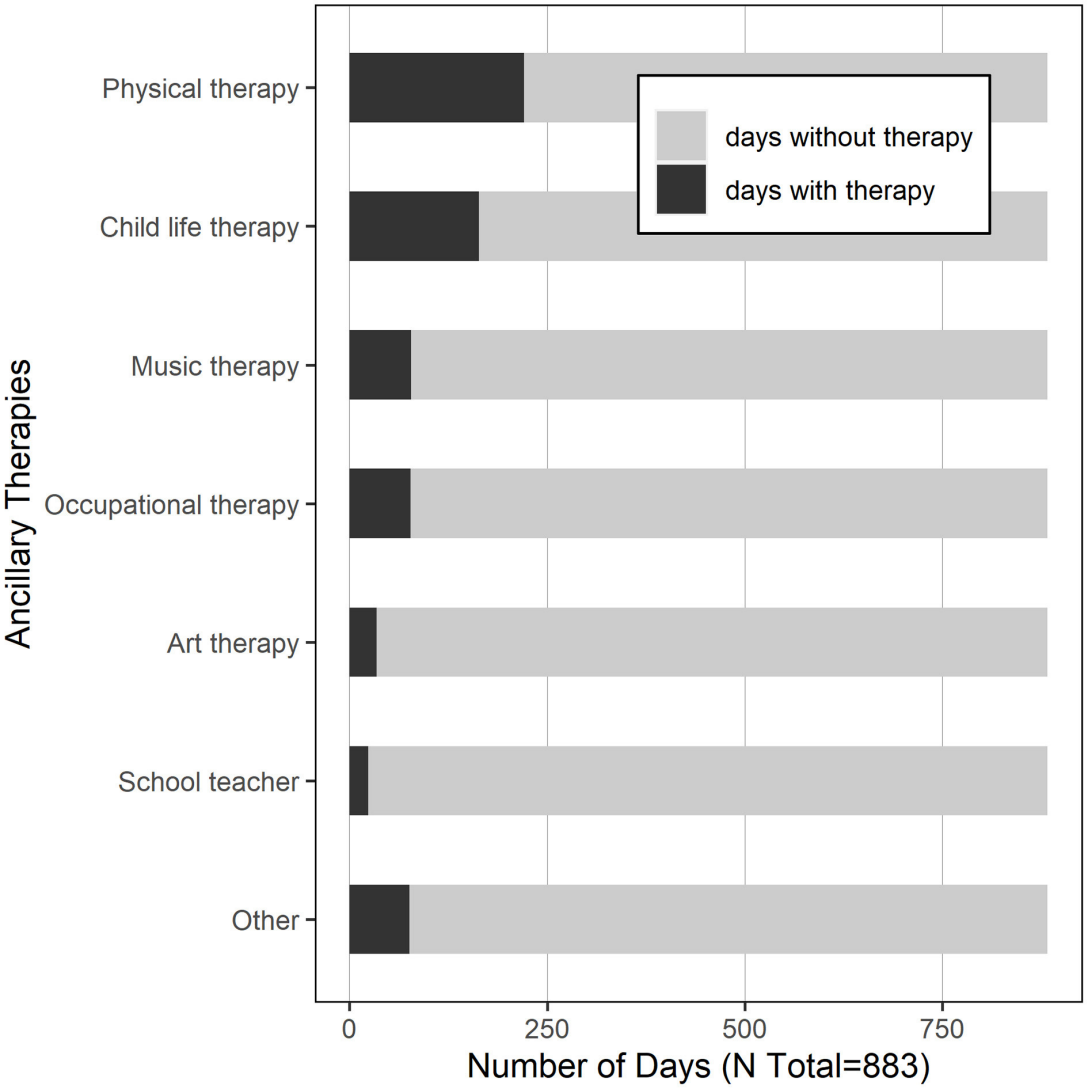
Hospital days with ancillary therapies provided.

A total of 883 hospital days were captured in this study, with a median of 10 days per subject (IQR 8–10). See Table 2 for the summary characteristics of these hospital days. In 93% of the days, the child was located on the transplant unit; in 7% of the days, children were located in the PICU. In 102 days (12%), children required supplemental oxygen. In 37% of the days, children were transfused with blood products. With respect to medication exposure, children received opiates in 60% of the study days, and benzodiazepines in 52%. Daily exposure to blood products and medications varied significantly by site (Figure 2). Children received physical therapy in 25% of the days, and occupational therapy in 9% of the days. Of note, in 425 days (48%), children received no ancillary therapies (Figure 3). A family member stayed with the child overnight in 89% of the study days.

**Table 2:** Bivariate associations with daily delirium (*n* = 883).

	**Overall (*n* = 883)**	**No delirium (*n* = 722)**	**Delirium (*n* = 161)**	***p*-value**
**Transplant day**				0.007
Median [IQR]	12 (3–25)	11 (3–23)	16 (6–31)	
**Location**				0.726
Transplant unit	818 (92.6%)	666 (92.2%)	152 (94.4%)	
PICU	62 (7.0%)	53 (7.3%)	9 (5.6%)	
Other	3 (0.3%)	3 (0.4%)	0 (0%)	
**Supplemental oxygen**				<0.001
Yes	102 (11.6%)	67 (9.3%)	35 (21.7%)	
No	781 (88.4%)	655 (90.7%)	126 (78.3%)	
**Blood product transfusion**				0.589
Yes	328 (37.1%)	265 (36.7%)	63 (39.1%)	
No	555 (62.9%)	457 (63.3%)	98 (60.9%)	
**Medication exposures in prior 24 h**				
Anticholinergics	555 (62.9%)	459 (63.6%)	96 (59.6%)	0.368
Antiepileptics	118 (13.4%)	85 (11.8%)	33 (20.5%)	0.005
Antipsychotics	98 (11.1%)	70 (9.7%)	28 (17.4%)	0.008
Benzodiazepines	463 (52.4%)	378 (52.4%)	85 (52.8%)	0.931
Melatonin	77 (8.7%)	52 (7.2%)	25 (15.5%)	0.002
Opiates	526 (59.6%)	424 (58.7%)	102 (63.4%)	0.288
Steroids	249 (28.2%)	173 (24.0%)	76 (47.2%)	<0.001
**Daily transplant therapy**				
Cyclosporine	192 (21.7%)	155 (21.5%)	37 (23.0%)	0.673
Defibrotide	136 (15.4%)	115 (15.9%)	21 (13.0%)	0.4
Eculizumab	10 (1.1%)	8 (1.1%)	2 (1.2%)	0.999
Methotrexate	24 (2.7%)	22 (3.0%)	2 (1.2%)	0.286
Mycophenolate Mofetil	146 (16.5%)	113 (15.7%)	33 (20.5%)	0.159
Sirolimus	48 (5.4%)	43 (6.0%)	5 (3.1%)	0.18
Tacrolimus	177 (20.0%)	130 (18.0%)	47 (29.2%)	0.002
Total body irradiation	4 (0.5%)	4 (0.6%)	0 (0%)	0.999

Delirium screening was successfully completed in more than 98% of the study days. In 13/883 days, delirium screening was missed; all 13 were at a single site on a single weekend.

### Delirium Frequency and Predisposing Risk Factors

Forty-eight children developed delirium over the course of the 10-day study, for a 45% occurrence rate. Among those children, a median of 2 days with delirium was noted (range 1–9). Delirium rates were higher in children under the age of 5 years (60 vs. 33% in older children, *p* = 0.008), in children who required supplemental oxygen (70% compared with 40%, *p* = 0.027), and in children exposed to steroids (60 vs. 34%, *p* = 0.016). Delirium rate in children with malignancies was no higher than in children with non-malignant diagnoses (41 vs. 56%, *p* = 0.2). Children transplanted for leukemia or lymphoma were less likely to become delirious than children transplanted for other indications (28 vs. 56%, *p* = *0.008*). There was no significant difference in delirium incidence among children who underwent allogeneic vs. autologous transplants in this cohort. In multivariable analysis, the only patient-level characteristic that was independently associated with delirium was younger age (median age for children with delirium was 3 years, compared with 4 years in children without delirium, *p* = 0.003). Older age was protective, with an adjusted odds ratio for a delirium diagnosis in children >5 years of 0.41 (CI 0.17–0.99) when compared with younger children.

### Daily Delirium Rate and Hospital Exposures

Children were diagnosed with delirium on 161/883 study days, for an overall delirium rate of 18% per day. Additionally, we looked at delirium rates only on the first calendar date captured (*n* = 84 subjects); point prevalence of delirium was 19%. When analyzed by site, daily delirium rates ranged from 0 to 42% (*p* <0.001), with a median of 16% (IQR 4.8–27.9%). Of note, 33% of the children who experienced delirium were only symptomatic during the overnight period and would have been missed with only once-daily screening.

**Figure 4:**
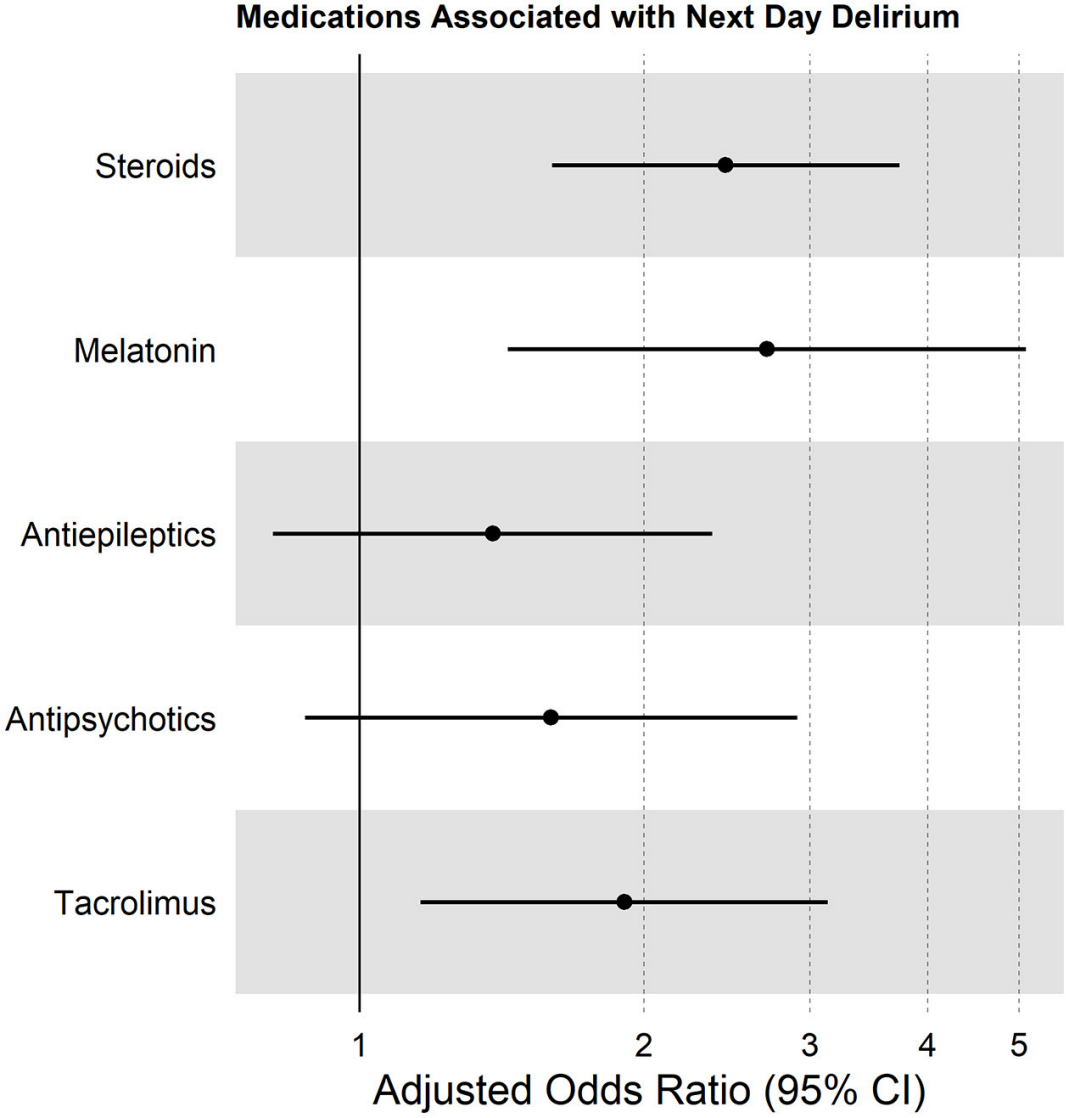
Forest plot showing multivariable analysis of medications associated with next-day delirium, accounting for within-site correlation, and controlled for patient age and need for supplemental oxygen.

Several medication categories were associated with next-day delirium in bivariate analyses: steroids, tacrolimus, antiepileptics, antipsychotics, and melatonin (Table 2). Children who were transfused blood products had a similar delirium rate (19%) in the 24 h after transfusion, compared with the 18% delirium rate in the non-transfused cohort (*p* = 0.63). In multivariable analysis, administration of steroids, tacrolimus, or melatonin were all independently associated with next-day delirium (Figure 4).

We noted a strong protective relationship with family member presence in the overnight period. Only 18% of the children who had a family member sleeping in their room developed delirium on the next day, compared with a 41% delirium rate in children without a family member present (*p* = 0.001).

## Discussion

### Delirium Prevalence

In this multi-institutional study, 45% of the children developed delirium during the peri-transplant period. This is consistent with delirium rates in adult populations undergoing hematopoietic cell transplants (>50%), and with small single-center pediatric studies that reported rates of 35–38% in children undergoing HCT (11, 12, 18, 22). Consistent with many other pediatric delirium studies, younger children experienced higher delirium rates (aOR 0.41 for children older than 5 years) (1–3, 5, 23, 24).

Children who required supplemental oxygen were more likely to be diagnosed with delirium, also consistent with prior pediatric delirium research (1, 5, 25). This may be a result of the underlying hypoxia and associated inflammation, which are known risk factors for delirium (26). On the other hand, the need for oxygen in this cohort may simply reflect the patient's underlying severity of illness. For example, a large prospective PICU study (*n* = 1,547) showed that severity of illness was independently associated with delirium risk, independent of need for respiratory support (5).

### Delirium Screening

Studies have shown that routine daily screening is needed to reliably detect delirium in hospitalized patients (27, 28). As described by Winsnes et al. in a single-center study, only 2.4% of the children were diagnosed with delirium in a hematology–oncology unit prior to implementation of routine delirium screening. Once daily screening began, delirium detection increased to 13% overall and 23% in children who had undergone a hematopoietic cell transplant (18). However, most pediatric oncologists remain unaware of their patients' risk for delirium. As a result of this critical knowledge gap, pediatric transplant units do not routinely screen for delirium, leading to missed or delayed diagnosis. This represents a lost opportunity to detect delirium early, when it is most amenable to therapeutic intervention (29).

Our data show that routine bedside delirium screening using the CAPD is highly feasible. Eleven different units, with different cultures and workflows, were able to successfully screen 106 children in >98% of the 883 study days. We also noted that delirium fluctuates over the course of the day-1/3 of our cohort only demonstrated delirium symptoms during the overnight period—so twice-daily screening is necessary in order to accurately capture the burden of delirium in this cohort.

### Iatrogenic Risk Factors for Delirium

In this pilot study, we describe associations between medication exposures and delirium, without implying any attributable causality. For example, we found an independent association between administration of steroids and development of delirium. It is likely that steroids are deliriogenic, as has been described in other studies (30). In contrast, although we noted a strong and independent association between receipt of tacrolimus and development of delirium, we cannot over-conclude based on this single study, since tacrolimus is often given as part of combination therapy with other agents that may themselves be delirium causing. Further research will be necessary to clarify this relationship.

With a point prevalence study design, we cannot establish definitive temporal relationships. For instance, we identified an independent association between administration of melatonin and a diagnosis of delirium. This is in contrast to a randomized placebo-controlled trial in a geriatric population that suggested that nightly ramelteon (a melatonin agonist) may provide protection against delirium (31). It is physiologically plausible that in our pediatric cohort, melatonin predisposed to hypoactive delirium, or it may be that melatonin was prescribed to promote sleep in children who were already experiencing delirium, as delirium in young children is known to cause significant circadian rhythm disruption (9). One cannot disentangle this chicken-and-egg phenomenon with the current study design. Nevertheless, despite this limitation, this point prevalence study provides important pilot data that are essential for the design of future large-scale longitudinal studies.

### Prescribing Practices

A large body of delirium literature demonstrates a strong and consistent relationship between benzodiazepines and pediatric delirium (1, 3, 5, 10, 22, 24, 32). In fact, a recent systematic review and meta-analysis described a pooled odds ratio of 3.5 for benzodiazepines and delirium in children (9). In contrast to the existing delirium literature, we did not find a significant relationship between benzodiazepine exposure and delirium in our cohort. This may reflect the overwhelming exposure to benzodiazepines in our subjects—only a minority of patients did *not* receive benzodiazepines at some time during the study. As we did not capture dosing or route of administration, we were not able to measure the strength of exposure in order to assess the relationship with delirium development. In addition to temporal relationships, future studies should focus on daily and cumulative dose exposures of medications of interest, as this may be an important area for intervention in order to decrease delirium burden.

Similarly, research has shown a dose-response relationship between red blood cell (RBC) transfusions and pediatric delirium (33). This pilot study was not designed to measure dose of RBC exposure (we described any level of exposure within 24 h and compared it with no exposure whatsoever). This will be important to study on a granular level in future research, as we hypothesize that there will be a dose-response effect between exposure to benzodiazepines and blood products and delirium incidence and duration.

As noted in Figure 2, benzodiazepine and transfusion practices vary widely between institutions. This is likely a function of provider choice rather than patient need and may present an opportunity to modify our peri-transplant practice in order to decrease delirium rates. Since our data also showed that delirium rates varied significantly between institutions, it is possible that unit-prescribing practices—a potentially modifiable factor—played a role in delirium risk.

### Non-pharmacological Approach to Delirium Prevention: Opportunities for Improvement

There is convincing evidence that delirium can be prevented by the use of a multi-component non-pharmacologic interventional protocol (8, 17, 34). In geriatric adults, an approach designed to address social isolation, physical immobility, and sleep disruption has been shown to decrease delirium rates by more than 50% (35). A single-center pediatric study showed a 39% decrease in PICU delirium rates with implementation of universal delirium screening, avoidance of deep sedation, and introduction of early mobilization (8). It is poignant that delirium rates in our study cohort were dramatically lower when parents remained in the child's room overnight. Conversely, it is striking that children received no ancillary therapies whatsoever on 48% of the study days. These represent possible areas for intervention: with increased physical and cognitive stimulation during the day, and enhanced family presence overnight, we may be able to decrease delirium rates in our at-risk population (36).

### Study Strengths and Limitations

This study has notable strengths. To date, it is the only multi-institutional study of pediatric delirium in HCT patients. With a mix of participating units from across North America, it generates a representative patient sample that is likely generalizable to the overall pediatric HCT population. A uniform definition was used for delirium, which was prospectively diagnosed, rather than relying on medical record review. The data set is very complete, with delirium status available for more than 98% of the study days.

However, this study also has important limitations. With a point prevalence study design, there is a very limited ability to account for temporality, and patients were followed at different time points in their peri-transplant course. This presents only a snapshot, as it does not take into account delirium that occurred before the 10 study days or delirium that developed afterward. Therefore, the 45% delirium rate we report is likely an underestimation of the true delirium burden in the peri-transplant period. In addition, since transplant units were self-selected to participate in this study, there is the possibility of bias—perhaps units already more attuned to delirium chose to participate. Also, the associations between certain medications and delirium status may be confounded by other associated aspects (for example, steroids are often used for engraftment syndrome, GVHD, etc., It is possible that rather than steroids, it is the engraftment syndrome itself that predisposes to delirium). Careful large-scale longitudinal research will be necessary to account for these complicated interactions. Finally, this pilot study was not designed to assess the effect of delirium on patient outcomes. This will be an important area to focus on in future studies.

### Conclusion

In conclusion, delirium occurred frequently in this cohort of children undergoing hematopoietic cell transplantation. Higher delirium rates were noted in children <5 years old and in association with specific medications. Delirium screening is highly feasible and necessary in this at-risk population. A large-scale prospective longitudinal study following children throughout their transplant course—from conditioning through engraftment—will be needed to fully describe the epidemiology of pediatric delirium in the peri-transplant period, and explore the effect of delirium on patient outcomes.

## Data Availability Statement

The raw data supporting the conclusions of this article will be made available by the authors, without undue reservation.

## Ethics Statement

The studies involving human participants were reviewed and approved by Weill Cornell Medical College Institutional Review Board. Written informed consent from the participants' legal guardian/next of kin was not required to participate in this study in accordance with the national legislation and the institutional requirements.

## Author Contributions

CT, LG, EM, JFi, JFr, MZ, BG, GS, and FB made substantial contributions to the study design, analysis, and interpretation of the data. KS, LB, YC, CD, CE, JFi, JFr, MH, CH, KM, JM, MiS, MaS, DW, MZ, and FB made substantial contributions to the acquisition and interpretation of the data and revised the manuscript for important intellectual content. CT drafted and revised the manuscript. All authors reviewed and approved the final version of this manuscript and agreed to be accountable for all aspects of the work.

## Conflict of Interest

The authors declare that the research was conducted in the absence of any commercial or financial relationships that could be construed as a potential conflict of interest.
